# Impact of a Scholarly Concentrations Program on Residents’ Scholarly Output and Professional Identity Formation

**DOI:** 10.7759/cureus.99641

**Published:** 2025-12-19

**Authors:** Rasika Behl, Clea Sarnquist, Rebecca Blankenburg, Hadassah Betapudi, Caroline Rassbach

**Affiliations:** 1 Pediatrics, Stanford University School of Medicine, Palo Alto, USA; 2 Pediatrics, Stanford University School of Medicine, Lucile Packard Children’s Hospital, Palo Alto, USA; 3 Epidemiology and Clinical Research, Stanford University School of Medicine, Palo Alto, USA

**Keywords:** career development, pediatrics, physician scientist, resident, scholarly activity

## Abstract

Background

Scholarly activity during residency helps build residents’ careers, establishes professional identity, supports the use of evidence-based medicine, and encourages the growth of future physician scientists. Furthermore, scholarship in residency is an important Accreditation Council for Graduate Medical Education requirement. Nonetheless, barriers to scholarship, such as limited time and challenges in finding mentorship, persist. To overcome these barriers, we implemented and evaluated a pediatric residency Scholarly Concentrations (SC) program, focusing on professional identity formation (PIF) and scholarly output. The SC program, which began in 2012, provides a longitudinal curriculum and faculty mentorship and support for residents’ scholarly projects. Residents choose among one of the following six areas of scholarly focus: basic science and translational research, clinical research, global health, community engagement and advocacy, quality and performance improvement, and medical education.

Methodology

In summer 2021, we emailed an anonymous electronic survey to all 175 pediatric resident alumni who completed the program and graduated between 2015 and 2020, asking questions related to key outcomes such as the impact of the SC program on professional identity and scholarly outputs during and since residency. Data were analyzed descriptively and with chi-squared tests.

Results

In total, 91 (52%) alumni completed the survey. Impact on PIF was high, with 69.2% (n = 63) reporting moderate/great impact on their careers, and 67.4% (n = 60) identifying research as important to their professional identity. Of respondents who were fellows or in university-based clinical practice at the time of the survey, 80.3% (n = 49) reported a moderate/great impact of the SC program on their career compared to 44.0% (n = 11) who were in non-university clinical settings (p = 0.03). Residency scholarship often translated to first-author articles, with 44.0% (n = 40) reported having first authorship on a manuscript related to their residency research. Post-residency, 76.9% (n = 70) of participants reported taking part in at least one type of research.

Conclusions

Our findings suggest that participation in the SC program was correlated with residents’ robust scholarly outputs, professional identities, and careers.

## Introduction

Engagement in scholarly activities during residency allows residents to integrate clinical and scholarly passions, innovate in children’s health, participate in career-building work outside of clinical work, and supports physician use of evidence in clinical practice [1]. Scholarship is also a required component of residency, with the Accreditation Council for Graduate Medical Education (ACGME) stating that “program[s] and faculty must create an environment that fosters the acquisition of such skills through resident participation in scholarly activities” (ACGME, 2020). In addition, the American Board of Pediatrics, American Medical School Pediatric Department Chairs, and the National Pediatric Scientist Collaborative Workgroup have all called for increased training and support for pediatric physician scholars and scientists, and improved on-ramps at different levels of training to enhance these pathways [2,3]. Finally, previous studies have shown that involvement in research during medical training serves as an entry point for a career in academic medicine or medical research [4,5].

Despite these requirements, recommendations, and the benefits of resident engagement in scholarship, challenges remain [6-8]. Key barriers identified in the literature include limited time away from clinical duties, insufficient education on research skills, and difficulty identifying and accessing mentors [9-11]. Nonetheless, there is evidence that these barriers can be addressed through formalized scholarship programs during residency [12]. Such programs, which often both mandate scholarship requirements and provide structure to meet those requirements, have resulted in up to a 10-fold increase in research productivity measured by the number of resident publications [13,14].

To support resident scholarship, the Stanford Pediatric Residency Program has had a Scholarly Concentrations (SC) program since 2012. All pediatric residents are required to participate in a minimum of three years of tailored, longitudinal curriculum and mentoring, culminating in the completion and presentation of a scholarly project. Residents choose one of six areas of scholarly focus, described below. The SC program aims to support residents’ scholarly growth, output, and professional identity as scholars.

Previous studies have focused primarily on short-term metrics such as publication counts; few have examined the long-term impact on graduates’ professional identity formation (PIF) or sustained engagement in research beyond residency. This study fills these gaps and is the first to assess the effects of the SC program on our graduates during and after residency. Further, as our program has been in place for over a decade, it can also serve as a model for newer programs of what a comprehensive SC program can achieve. We grounded this evaluation in PIF theory [15,16] with a primary aim to assess the impact of the SC program on graduates’ PIF and scholarly outcomes.

This article was previously presented as a meeting abstract at the 2024 Association of Pediatric Program Directors (APPD) Annual Spring Meeting on April 17, 2024.

## Materials and methods

Educational intervention: Scholarly Concentrations program

The SC program began in 2012, aiming “to develop physicians who approach medicine in a scholarly way and incorporate scholarship into their careers to improve children’s health.” The SC program incorporates a broad definition of scholarship, including discovery, integration, application/engagement, and/or teaching [17]. To support this definition and meet the diverse interests of the residents, six concentration areas are defined. The six SCs are basic science and translational research, clinical research, global health, community engagement and advocacy, quality and performance improvement, and medical education.

Each concentration has two to four designated SC leaders who teach and facilitate scholarly educational sessions and mentor residents in their projects, sometimes in coordination with additional subject expertise mentor(s). The SC leaders are faculty and staff members in the Department of Pediatrics who receive approximately 10% salary support for this educational role. Residents can apply for up to $3,000 in research grant funding and up to $2,000 in travel funding to support their research project.

The SC program includes a longitudinal curriculum. Each resident attends two or three educational scholarship “retreats” per year, for a total of 11-12 retreats across all three years of residency. This curriculum is designed to meet the educational needs of each cohort as they move through the scholarship process (for example, literature search guidance in the middle of PGY1, and presentation skills early in PGY3) (Table 1). Research retreats are held in the afternoon for five hours each, including lunch and social time, and clinical coverage is provided to ensure residents’ participation. Most retreats are structured to include half of each residency class at a time to enhance smaller-group learning, and each retreat includes structured research skills learning as well as 1.5 hours of dedicated time with SC leaders for residents to discuss and troubleshoot their scholarly projects.

**Table 1:** Scholarly Concentration topics and time spent per topic by resident year.

Topic	Year	Time devoted
Research resources and funding	All	~1 hour per year
Intro to quality improvement	PGY1	1 hour
Addressing health disparities and racism in research planning, implementation, and results sharing	All	1 hour per year, 3 hours total
Writing a good research question	PGY1	30 minutes
Quality literature searches	PGY1	1 hour
Mentor-mentee relationships	PGY1	1 hour
Data analysis (separate qualitative and quantitative sessions)	PGY2	1 hour
Career development (separate fellowship and non-fellowship career sessions)	PGY2	1 hour
Communicating research results	PGY2	1 hour
Abstract writing	PGY3	1 hour
Verbal presentation skills	PGY3	1 hour
Creating an effective poster	PGY3	1 hour
Curriculum improvement feedback sessions	PGY2 and 3	30 minutes per year

In additon to the general scholarship retreats for all residents, residents also participate in a specific educational “block rotation” for their individual concentration that lasts between one and four weeks, depending on the concentration, during their PGY2 year. In that block rotation, residents learn about research methods and approaches specific to their chosen area of concentration and have dedicated time to work on their study design, institutional review board application, and analysis and dissemination plans.

All pediatric residents are required to present their scholarly work as a poster at our Department’s Research Day during their PGY3 year. Although not required, many residents also present at regional or national conferences.

Study design

We performed a retrospective, cross-sectional, anonymous, survey-based study in the summer of 2021 using Qualtrics (Provo, UT). We distributed the survey to 175 pediatric alumni who had completed the full SC program, with the first cohort of residents who had the full program graduating in 2015 and the most recent cohort graduating in 2020. We distributed the survey via email with a unique link to each graduate with five follow-up reminder emails, every one to three weeks over a three-month period. The Stanford University Institutional Review Board (IRB) reviewed the study design and determined that this project did not meet the definition of human subjects research.

Measures

Survey questions were developed de novo using PIF theory as a conceptual framework, with particular focus on constructs related to professional competence in scholarship, socialization and belonging, professional roles and responsibilities, and meaning and purpose. Questions were developed and adapted by our SC and residency leadership teams, guided by the literature on pediatrics residency scholarship program evaluation surveys from across the country, as well as PIF-related research [12,18], for content validity. The PIF framework seeks to elucidate and connect the factors that contribute to one’s professional identity, such as having dedicated mentors.

The survey included a mixture of multiple-choice, Likert-scale, and dichotomous yes/no questions with follow-up open-ended questions to allow respondents to provide clarity and details. It was pilot-tested for response process validity with five current residents, who volunteered, and the survey was revised, taking their feedback into account, before deploying [19].

Key outcomes included the impact of the SC program on professional identity and scholarly outputs during and since residency. We asked whether alumni of our program believed that the SC program prepared them for their careers; to what extent they have been able to integrate scholarly pursuits into their careers; if their career goals changed because of participation in the SC program; and what their scholarly output has been during and since residency. Survey questions also included demographics, perceived usefulness of the program, career path, additional degree information, and data on research funding received. The survey also included opportunities for open-ended responses (see the Appendix for the complete survey).

Data analysis

Surveys were excluded from analysis if fewer than 50% of the questions were completed (six surveys were ultimately excluded). Quantitative data were analyzed descriptively and with chi-squared tests, with a significance threshold of <0.05, in Stata SE 14.2 (Stata Corp., College Station, TX, USA). Multiple-response and “other” question responses were reviewed and categorized into pre-existing categories or new variable categories. Qualitative open-ended question data were reviewed, and illustrative quotes were extracted and used to clarify quantitative results.

## Results

Respondents

Of the 91 surveys analyzed, all but two surveys were 100% complete; those two incomplete surveys were 70% complete. Response rate was 52%, with 91/175 alumni participating.

Post-residency paths varied, with 40 (69.0%) reporting starting a fellowship program post-residency and 16 (27.6%) doing a chief residency. At the time of the survey, 31.8% (n = 29) of respondents were fellows, 22.0% (n = 20) were in non-university-based clinical practice, and 35.2% (n = 32) were in university-based clinical practice.

Distribution of responses across graduation years was fairly even (Table 2); 44% of the class of 2019 participated in the survey, 48% of the classes of 2017-2018 participated, 52% of the class of 2020, 58% of the class of 2015, and 71% of the class of 2016 (data not shown). Respondents predominantly identified as women, and the majority identified as non-Hispanic White, followed by Asian (Table 2); in the full alumni population, 79.6% of residents identified as women, 56.3% identified as Caucasian, and 28.4% identified as Asian (data not shown). Response rates by concentrations were also similar to the distributions of residents across concentrations within our program (data not shown).

**Table 2:** Sociodemographic characteristics of pediatric resident alumni study participants. *: Includes participants who identify as multiracial, where one or more of their racial identities are considered URM (underrepresented minority), defined by the Association of American Medical Colleges as “racial and ethnic populations that are under-represented in the medical profession relative to their numbers in the general population” [20]. **: Includes participants who identify as multiracial, where none of their racial identities are considered URM.

All participants, N = 91 (%)	
Gender identity	
Woman	78 (85.7%)
Man	13 (14.1%)
Other/Prefer not to state	0
Race/Ethnicity	
Asian	17 (18.5%)
Black	1 (1.1%)
Hispanic	3 (3.3%)
Non-Hispanic White	55 (60.4%)
2+ URM*	6 (6.5%)
2+ non-URM**	2 (2.2%)
Year of residency graduation	
2015	15 (16.3%)
2016	19 (20.9%)
2017	15 (16.3%)
2018	13 (14.1%)
2019	12 (13.0%)
2020	17 (18.5%)
Scholarly Concentration	
Medical education	30 (32.6%)
Clinical research	25 (27.5%)
Community engagement and advocacy	18 (19.6%)
Basic and translational science	7 (7.6%)
Quality and performance improvement	7 (7.6%)
Global health	4 (4.4%)

Effect on career

Overall, survey participants reported that the SC program had a significant impact on their careers, with 69.2% (n = 63) reporting moderate or great career impact, and 69.2% (n = 63) reporting that the SC program changed their understanding of academic medicine. Almost half (49.5%, n = 46) of the respondents reported that the SC program changed or confirmed their post-residency career goals, and 56.0% (n = 51) reported that the structure of the SC program was very or extremely flexible in allowing them to achieve their individual goals. Furthermore, 80.3% (n = 49) of respondents who were fellows or in university-based clinical practice at the time of the survey reported a “moderate” or “great” impact of the SC program on their career compared to only 44.0% (n = 11) who were in non-university clinical settings and reported a moderate/great impact of the program (p = 0.03).

Professional identity formation

Scholarship played an important role in many of the respondents’ professional identities. Post-residency, 76.9% (n = 70) of participants reported taking part in at least one type of scholarship. More than two-thirds of respondents (67.4%, n = 60) identified at least one type of research as being one of the most important aspects of their professional identity; specifically, 20.7% identified medical education and advocacy research as important, and 19.7% identified advocacy research as important (respondents could choose more than one option). Furthermore, 40.2% (n = 35) of participants strongly agreed that research was useful for their career. Clinical medicine and clinical education/teaching were also key aspects of participants’ professional identities (Table 3).

**Table 3:** Aspects of current career most important to professional identity (could choose more than 1). *: Determined by participants selecting options in a multiple-choice, “select all that apply” survey question.

Career aspects with importance to professional identity*	N	%
Clinical Medicine	89	96.7
Clinical Education/Teaching	65	70.7
Research	60	67.4
Administration	13	14.1

Participants reported that the SC program and publication of a manuscript on research conducted during residency had a significant impact on their professional identity. Specifically, 78% of participants (n = 47) who reported a “moderate” or “great” impact of the SC program on their career development also identified research as one of the key parts of their professional identity compared to 22% (n = 13) of participants who reported a “minor” impact of the SC program on their career (p = 0.005). Furthermore, 83% of participants (n = 40) who reported publishing a manuscript identified research as one of the most important parts of their professional identity compared to 17% (n = 8) who published a manuscript and did not identify research as one of the most important parts of their professional identity (p < 0.001). Similarly, 75% of participants (n = 36) who reported publishing a manuscript identified research as “significantly” or “moderately” impacting their professional identity compared to 25% (n = 12) who published a manuscript and identified research as “somewhat,” “slightly,” or “not impacted” their professional identity (p < 0.001).

There was also an association between professional identity and current employment. Oberall, 83.6% (n = 46) of participants who identified research as one of the most important parts of their professional identity were fellows or in university-based clinical practice at the time of the survey compared to 16.4% (n = 9) of participants who identified research as one of the most important parts of their professional identity and were in non-university-based clinical practice (p = 0.001).

Scholarly outputs

Reported authorship was high, with 44.0% (n = 40) of respondents having been a first author on a manuscript for research undertaken during residency. Participants also reported sharing research results during residency through oral or poster conference presentations, academic workshops, and contributions to medical books and book chapters (Table 4).

**Table 4:** Academic products resulting from scholarly projects started during residency (2015-2020).

	Yes (number of products)				No
	Total N(%)	1	2	3+	Total, N (%)
Peer-reviewed manuscripts (first author) (n = 91)	40 (44%)	24 (26%)	10 (11%)	6 (7%)	51 (56%)
Peer-reviewed manuscripts (not first author) (n = 90)	35 (39%)	23 (26%)	5 (6%)	7 (8%)	55 (61%)
Oral or poster presentation for national or international conferences or meetings (n = 91)	68 (75%)	28 (31%)	12 (13%)	28 (31%)	23 (25%)
Workshop at academic or continuing education meetings (n = 90)	24 (27%)	8 (9%)	6 (7%)	10 (11%)	66 (73%)
Medical book or book chapter (n = 90)	13 (14%)	7 (8%)	3 (3%)	3 (3%)	77 (86%)
Medical or book review (n = 90)	10 (11%)	5 (6%)	3 (3%)	2 (2%)	80 (89%)
Letter to the editor (n = 89)	2 (2%)	1 (1%)	1 (1%)	0 (0%)	87 (98%)

We also asked participants if they continued publishing peer-reviewed articles after residency, unrelated to their publishing during residency. More than half of participants (53.3%, n = 48) reported publishing a manuscript post-residency. Those who were fellows or in university-based clinical practice at the time of the survey were significantly more likely to have published a manuscript after graduation (78%, n = 39) compared to those in non-university-based clinical practice (4%, n = 2) (p = 0.001).

Grant funding

Many participants reported seeking funding to support their research and scholarship after graduation from residency. Specifically, 44.0% (n = 40) of participants reported applying for any funding post-residency. This included internal funding from their home institution, National Institutes of Health (NIH) funding, private foundation grants, and pharma/biotech funding. About a third of participants (n = 28) reported receiving internal grant funding; of these, 21 were funded as principal investigator (PI), and 7 as a co-investigator. Two participants reported receiving NIH career development grants since residency, one of whom also reported receiving NIH research grant funding. Four other participants were also funded for an NIH research grant, one as a PI and the remaining three in another role.

## Discussion

These results highlight how our graduates identify the SC program as important in supporting both their professional identity formation as scholars as well as their scholarly output during residency and beyond, especially among those who pursued academic medicine. We also found a significant association among participants reporting that the SC program impacted their career development, participants identifying research as a key element of their professional identity, and participants publishing a manuscript. While these data do not allow us to tease out if those who entered residency interested in a career that included research engaged more with the SC program and were more likely to publish, or if the SC program supported residents to incorporate scholarship more in their careers and publish, or both, our findings suggest the SC program is achieving its goals in supporting professional development in research. We suspect that research success may be supported by our SC program and that this success could help residents without a prior interest in research and scholarship become more interested in this path.

Our findings that the SC program supports PIF as a scholar are important, as our SC program supports a broad definition of scholarship, allowing an additional on-ramp into becoming a physician scholar after medical school. Our findings align with previous research that shows that structured, supportive residency research programs support PIF. Interestingly, this connection has been shown to be true both with programs that use a more traditional definition of research as well as those that use a broad definition, as we do, including scholarly approaches to fields such as medical education and advocacy [21-24]. Our current study builds upon prior research in showing how scholarly concentrations can be a strategy for increasing the physician scholar, including the physician scientist, pipeline.

As it remains uncommon to have a residency program-level SC program, particularly one with a broad definition of scholarship, our findings fill a gap in understanding how such a program and scholarship requirement affect resident scholarly engagement and output. Data from other institutions focused on resident scholarship are consistent with our findings on scholarly output. The University of Hawai’i implemented a pediatric residency research requirement in 2001 and found that its residents’ scholarly output, measured by authorship on manuscripts, increased 10-fold [13]. The scholarly concentration program provides a promising way of addressing the leaky pathway of pediatric physician scholars and scientists. We believe that training residents in scholarship and supporting their research success may improve their research motivation and enjoyment and enhance their professional identity as scholars. As such, programs such as ours have the potential to prepare graduates to continue pursuing child health research, be competitive in gaining research funding, and advance children’s health.

Limitations

There are several limitations to this study. The first limitation was the response rate; while 52% felt meaningful, especially as the data were collected during the COVID-19 pandemic, we would have liked to have a better response rate, and our results may have been impacted by non-response bias. In particular, graduates who felt scholarship was important may have been more likely to complete the survey (for example, the subgroup of participants in non-university clinical practice had a smaller n relative to participants in university-based clinical practice and/or fellowship), and we cannot compare the demographics of those who did, and did not, complete the survey as the survey was done anonymously. An important follow-up will be a future prospective study tracking residents over time to better understand the causal relationship between this program and scholarly outcomes. Second, there might have been social desirability bias in answering the survey, which we attempted to address by keeping the survey anonymous. Third, as mentioned above, the survey was administered in 2021 during the pandemic. It is possible that this reduced our response rates due to competing demands and provider burnout, which has been widely documented. Of note, provider burnout would have likely biased participants not to report the positive effects of the program, toward the null. Lastly, because all residents participate in the SC program, and we did not have historical data, it was not possible to have a comparison group.

## Conclusions

Our findings indicate that our SC program for pediatric residents had a considerable positive impact on graduates’ professional identities as scholars and their scholarly outputs. In addition, we found associations among graduates reporting that the SC program had an impact on their careers, graduates publishing, and graduates identifying scholarship as important to their professional identity. These results bolster previous findings suggesting that having a structured program supporting resident scholarship supports residents’ PIF and improves scholarly outputs, including published papers and funding. We propose future research to query if and how scholarship during residency supports an adaptive learning mindset, professional fulfillment, and physician wellness. We also hope to delve further into barriers to engaging in scholarship, especially for groups traditionally underrepresented in academic medicine and research careers.

## Appendices

**Table 5:** Full survey. Credits: Rasika Behl, Clea Sarnquist, Carrie Rassbach, and Rebecca Blankenburg.

Alumni Survey 2021		
Section	Question	Response options
Introduction		Alumni Survey 2021 Hello! As alumni of the Stanford Pediatrics Residency Program, we seek your input to help evaluate the Stanford Pediatric Residency Program's Scholarly Concentrations program. This confidential survey is being distributed to all alumni from the graduating class of 2015 onwards. Results will be analyzed in aggregate to ensure confidentiality. This project has received IRB approval at Stanford University. Our aim is to use this data to understand how the Scholarly Concentrations program has affected the career paths of graduates. The entire survey should take no more than 15 minutes to complete. You will have the option to save your responses and come back later to finish the survey if you wish. We appreciate your time and your thoughtful responses! -Clea Sarnquist, Director of the Scholarly Concentrations Program -Rasika Behl, Program Officer, Scholarly Concentrations Program -Carrie Rassbach, Pediatrics Residency Program Director -Rebecca Blankenburg, former Pediatrics Residency Program Director
Demographics	What Scholarly Concentration were you in?	Basic/Translational Science (1) Clinical Research (2) Community Engagement and Advocacy (StAT program) (3) Medical Education (4) Quality and Performance Improvement (5) Global Health (6) I don’t know (7)
Demographics	What year did you graduate from residency?	2015 (4) 2016 (5) 2017 (6) 2018 (7) 2019 (8) 2020 (9)
Demographics	What residency program were you in?	Categorical Pediatrics (1) Combined Program (Peds-Anesthesia, Child Neuro, Peds-Genetics) (2)
Demographics	What degrees do you currently hold? Please select all that apply.	MD/DO (1) PhD (2) JD (3) MPH (4) MBA (5) MEd or MHPE (6) MS (including MSc, MSCE, MSHP, etc.) (7) Other Masters’ or Doctoral Degree (please specify in the field below) (8) ________________________________________________
Demographics	Your gender (select all that apply):	Woman (1) Man (2) Transgender (8) Gender non-conforming (5) Gender non-binary (6) Genderqueer (7) Other: (3) ________________________________________________ Prefer not to disclose (4)
Demographics	Your race/ethnicity (select all that apply):	American Indian or Alaska Native (1) Asian (2) Black or African American (3) Hispanic or Latino (4) Native Hawaiian or Other Pacific Islander (5) White (6) Other (99) ________________________________________________ Prefer not to disclose (98)
Scholarly Concentration Program	To what extent did the Scholarly Concentration program impact your career?	To a great extent (5) To a moderate extent (4) To a minor extent (3) No impact (2)
Scholarly Concentration Program	To what extent did the Scholarly Concentration program impact your career? = No impact Please tell us more:	Program not useful (1) I don’t do research (2) Other: (3) ________________________________________________
Scholarly Concentration Program	Did the Scholarly Concentration Program change your understanding of academic medicine?	Yes (1) No (2)
Scholarly Concentration Program	Did the Scholarly Concentration Program change your understanding of academic medicine? = Yes How did it change your understanding of academic medicine?	_____________________________________________________
Scholarly Concentration Program	Did participation in the Scholarly Concentration Program change or confirm what you chose to do after residency?	Yes (1) No (2) Unsure (3)
Scholarly Concentration Program	Did participation in the Scholarly Concentration Program change or confirm what you chose to do a... = Yes Please let us know HOW and WHY the SC Program may have changed (or confirmed) your original primary career goal.	_____________________________________________________
Scholarly Concentration Program	Did participation in the Scholarly Concentration Program change or confirm what you chose to do a... = No Or Did participation in the Scholarly Concentration Program change or confirm what you chose to do a... = Unsure Please explain how the Scholarly Concentration program has or has not impacted your career:	_____________________________________________________
Scholarly Concentration Program	As a result of your Scholarly Concentrations project, as well as any other scholarly projects you started during residency, how many of the following academic products have you had?	0 (1) 1 (2) 2 (3) 3+ (4) Peer-Reviewed Manuscripts (First Author) (1) Peer-Reviewed Manuscripts (Not First Author) (2) Oral or Poster presentation for National or International Conference or Meeting (3) Workshop at academic or continuing education meetings (4) Medical Book or Book Chapter (5) Medical Review (6) Letter to the Editor (7) Book Review (8) Other type of publication (please specify) (9)
Scholarly Concentration Program	Which of the following statements best describes the level of flexibility offered in the SC program to allow you to achieve your individual goals?	Not at all flexible (0) Slightly flexible (1) Moderately flexible (2) Very flexible (3) Extremely flexible (4)
Alumni Career Path	After graduating from the pediatrics residency program, did you do any of the following?	2nd Residency (2) Chief Residency (3) Fellowship (4) Other training program (1) None of the above (5)
Alumni Career Path	After graduating from the pediatrics residency program, did you do any of the following? = Fellowship If you went on to a fellowship, in what sub-specialty?	________________________________________________________________
Alumni Career Path	What is your current role? Please check all that apply:	Fellow (1) Chief Resident (2) Clinical Practice, not university-based (3) Clinical Practice, university-based (4) NIH (5) Federal Agency, other than the NIH (please specify) (6) Research Institute, Non-federal (please specify) (7) Pharmaceutical/Biotech Industry (8) Consulting/Law/Finance (9) Other (please specify) (10) ________________________________________________
Alumni Career Path	In your current position, please estimate how you divide your time among the following responsibilities, in % time spent per month:	Clinical/Patient Care : _______ (1) Research : _______ (2) Teaching : _______ (3) Administration : _______ (4) Other (Please Specify) : _______ (5) Total : ________
Alumni Career Path	Which, if any, of the following types of research have you done after residency? Please check all that apply:	Global Health (1) Community Engagement/Advocacy (2) Medical Education (3) Quality Improvement (4) Clinical Research (5) Basic/Translational Science (6) Health Services Research (7) Other (please specify): (8) ________________________________________________ None (9)
Alumni Career Path	Which, if any of the following types of publications that you have authored or co-authored since residency? Please check all that apply.	Abstracts (for National or International Conference or Meeting) (1) Medical Book or Book Chapter (2) Medical Review (3) Medical Manuscript / Original Research (4) Letter to the Editor (5) Book Review (6) Other type of publication (please specify) (7) ________________________________________________ None (8)
Alumni Career Path	To what extent has research impacted your professional identity?	Significantly impacted my professional identity (1) Moderately impacted my professional identity (2) Somewhat impacted my professional identity (3) Slightly impacted my professional identity (4) It has not impacted my professional identity (5)
Alumni Career Path	Which aspects of your career are most important to your professional identity? Please select all that apply	Clinical medicine (1) Clinical education/teaching (2) Administration (3) Quality Improvement/Performance Improvement research (4) Global Health research (5) Community Engagement/Advocacy research (6) Health policy research (7) Basic/Translational science research (8) Health Services research (9) Medical Education research (10)
Alumni Career Path	The following statements refer to some aspects of educational research. Please answer all the questions sincerely. Slide the bar to one of the numbers next to each of the statements below. By selecting number 1 you indicate that you strongly disagree. By selecting number 7 you indicate that you strongly agree.	Strongly Disagree Agree 1 2 3 4 5 6 7 Research is useful for my career The skills I have acquired in research will be helpful to me in the future Research should be indispensable in my professional training
Alumni Career Path	Following completion of your residency, have you ever applied for research funding of any kind?	Yes (1) No (2)
Alumni Career Path	If Following completion of your residency, have you ever applied for research funding of any kind? = Yes Please indicate all research funding you received following completion of your residency program. Check all that apply:	Funded as PI (1) Funded in other role (2) Internal grant (1) NIH - Mentored Career Development Awards (2) NIH - Research Grant (3) Other Federal Grants (4) PCORI/AHRQ (5) Private Foundation (6) Pharma/Biotech (7) Other (please specify) (8)
Alumni Career Path	Please write anything else you’d like to tell us about the Scholarly Concentration program below:	________________________________________________________________ ________________________________________________________________ ________________________________________________________________ ________________________________________________________________ ________________________________________________________________
